# Extracellular cyclic adenosine monophosphate‐dependent protein kinase A autoantibody and C‐reactive protein as serum biomarkers for diagnosis of cancer in dogs

**DOI:** 10.1111/vco.12450

**Published:** 2018-11-28

**Authors:** Min‐Ok Ryu, Byung‐Gak Kim, Ul‐Soo Choi, Kwan‐Hyuck Baek, Young‐Ki Song, Qiang Li, Kyoung‐Won Seo, Sandra Ryeom, Hwa‐Young Youn, Dong‐Ha Bhang

**Affiliations:** ^1^ Laboratory of Veterinary Internal Medicine College of Veterinary Medicine, Seoul National University Seoul Republic of Korea; ^2^ Department of Molecular and Cellular Biology Sungkyunkwan University School of Medicine Suwon Republic of Korea; ^3^ Department of Veterinary Clinical Pathology and Bio‐safety Research Institute College of Veterinary Medicine, Chonbuk National University Jeonju Republic of Korea; ^4^ Biattic Corporation Suwon Republic of Korea; ^5^ College of Veterinary Medicine Chungnam National University Daejeon Republic of Korea; ^6^ Department of Cancer Biology Perelman School of Medicine at the University of Pennsylvania Pennsylvania; ^7^ BK21Plus Program for 21st Century Biomedical Science Leader Development Sungkyunkwan University School of Medicine Suwon Republic of Korea

**Keywords:** autoantibody, biomarker, cancer, C‐reactive protein, extracellular protein kinase A

## Abstract

Protein kinase A, a cyclic adenosine monophosphate (AMP)‐dependent enzyme, normally exists within mammalian cells; however, in cancer cells, it can leak out and be found in the serum. Extracellular cyclic AMP‐dependent protein kinase A (ECPKA) has been determined to increase in the serum of cancer‐bearing dogs. However, there have been no reports in the veterinary literature on serum ECPKA autoantibody (ECPKA‐Ab) expression in dogs with cancer. The aim of this study was to evaluate ECPKA‐Ab and C‐reactive protein (CRP) as serum biomarkers for cancer in dogs. ECPKA‐Ab and CRP levels were detected by an enzyme‐linked immunosorbent assay in serum samples from dogs with malignant tumours (n = 167), benign tumours (n = 42), or non‐tumour disease (n = 155) and from healthy control dogs (n = 123). ECPKA‐Ab and CRP levels were significantly higher in the dogs with malignant tumours than in those with benign tumours or non‐tumour diseases, as well as in the healthy controls (*P* < 0.001, Kruskal‐Wallis test). There was a significant positive correlation between the neoplastic index, which was developed using ECPKA‐Ab and CRP levels, and the presence of cancer in dogs (*P* < 0.001); the area under the receiver‐operating characteristic curve was estimated to be >0.85 (*P* < 0.001). In conclusion, ECPKA‐Ab is a potential serum biomarker for a broad spectrum of cancers. Combined measurement of CRP and ECPKA‐Ab levels in serum improves the sensitivity and accuracy of a diagnosis of cancer in dogs.

## 1 INTRODUCTION

An early cancer diagnosis is important not only in humans^1^, ^2^ but also in companion animals,^3^ and can lead to better quality of life and longevity by allowing early and appropriate treatment.^1^, ^2^ However, most cancers in dogs are detected by imaging or histopathology when it is too late to treat them.

Protein kinase A (PKA) is a cyclic adenosine monophosphate (AMP)‐dependent enzyme that participates in the proliferation, differentiation, metabolism and apoptosis of cells in mammals.^4^ PKA can leak out of cancer cells; when this occurs, it is known as extracellular cyclic AMP‐dependent protein kinase A (ECPKA).^5^ ECPKA has been found to be higher in serum samples from human patients with cancer than in those from individuals without cancer, suggesting its potential as a powerful diagnostic marker of cancer in humans.^5^, ^6^, ^7^, ^8^ In a previous study, we demonstrated a significant elevation in ECPKA levels in serum samples from dogs with cancer and suggested that ECPKA could be an important candidate diagnostic biomarker of canine malignancy.^9^ However, although ECPKA is a powerful predictor of canine cancer, it is a fragile enzyme in certain situations. For example, it has only 20% activity after two freeze‐thaw cycles.^6^ Therefore, detection of ECPKA autoantibodies (ECPKA‐Ab) in serum has been investigated for many years in human medicine, and it has been demonstrated that an elevated serum ECPKA‐Ab level has better diagnostic value for cancer than ECPKA alone.^10^ However, as of yet, there have been no reports in the veterinary literature on serum ECPKA‐Ab expression in dogs with cancer. Therefore, we have been interested in whether the serum ECPKA‐Ab level could be a stable and accurate diagnostic biomarker of cancer in dogs.

C‐reactive protein (CRP) is well known to be secreted in the acute phase of inflammation and is also a marker of cancer.^11^, ^12^ CRP levels have been shown to be significantly higher in human patients with colorectal, lung, prostate and breast cancers than in controls.^13^, ^14^, ^15^, ^16^ Various hypotheses have been put forward to explain why inflammation is a risk factor for cancer and why it occurs when cancer progresses.^16^ Several veterinary oncology researchers have suggested that elevation of serum CRP is not necessarily diagnostic of cancer in dogs,^17^, ^18^ even though CRP expression has been found to be higher in dogs with malignancy than in those without the disease and to indicate the stage of cancer progression.^19^, ^20^ However, Selting et al. demonstrated that the serum thymidine kinase 1 activity and CRP levels in the serum of dogs with cancer have high diagnostic value as markers of canine malignancy.^21^, ^22^, ^23^ They showed that the thymidine kinase 1 activity was high in dogs with hemangiosarcoma and other malignant canine tumours and that a combination of thymidine kinase 1 activity and CRP expression levels had increased the diagnostic accuracy for detection of cancer. Therefore, CRP might be used as an adjunct to other serum biomarkers to improve our ability to diagnose cancer in dogs.

The objectives of this study were to evaluate the diagnostic significance of ECPKA‐Ab in canine malignancy and to determine if combined measurement of CRP and ECPKA‐Ab levels in serum improves diagnostic accuracy for detection of cancer in dogs.

## 2 MATERIALS AND METHODS

### 2.1 Animals and samples

Four hundred and eighty‐seven privately owned dogs (123 control dogs with no known disease, 42 with benign tumours, 167 with malignant tumours, and 155 with non‐tumour disease) were included in the study. Serum samples were provided by Seoul National University Veterinary Medical Teaching Hospital and several local animal clinics in the Republic of Korea. All of the dogs included in this study underwent a physical examination; blood examination including complete blood counts, serum biochemical profiles and electrolytes; and diagnostic imaging such as abdominal sonography, radiography (thoracic and abdominal) and an optional echocardiogram or computed tomography. Malignant tumours were diagnosed by cytology or/and histopathology. One hundred and twenty‐three controls with no known disease visited the animal clinic for neutralization or regular checkup and were confirmed to have no abnormalities based on medical examination. All experiments were approved by and followed the policies and regulations of the Laboratory Animals Institutional Animal Care and Use Committee (SNU‐180130‐2; Seoul National University, Seoul, Korea).

### 2.2 ECPKA autoantibody ELISA

The presence and level of ECPKA‐Ab in canine serum samples were assessed by enzyme‐linked immunosorbent assay (ELISA). First, 100 μL of canine serum samples diluted 500‐fold in reagent diluent were added to 96‐well ELISA plates pre‐coated with canine PKA Cα and incubated for 1 hour at room temperature. The plates were then incubated further with anti‐dog immunoglobulin G:horseradish peroxidase antibodies for 1 hour at room temperature in a dark room. Next, the plates were developed with 3,3′,5,5′‐tetramethylbenzidine substrate solution for 15 minutes at room temperature. The reaction was stopped with 50 μL of stop solution. Absorbance was determined at 450 nm using a scanning multi‐well spectrophotometer (Molecular Devices, Sunnyvale, California).

### 2.3 Canine CRP ELISA

The serum CRP concentration was assessed using a canine CRP ELISA kit (Abcam, Cambridge, UK) following the manufacturer's instructions. Briefly, 100 μL of canine serum samples diluted in reagent diluent were added to 96‐well ELISA plates pre‐coated with anti‐canine CRP antibodies and incubated for 10 minutes at room temperature. After washing, 100 μL of the enzyme‐antibody conjugate was added to the plates, followed by incubation for 10 minutes at room temperature in the dark. The plates were then developed with 3,3′,5,5′‐tetramethylbenzidine substrate solution for 5 minutes at room temperature, and the reaction was stopped with 100 μL of stop solution. Absorbance was determined at 450 nm with a scanning multi‐well spectrophotometer (molecular devices).

### 2.4 Statistical analysis

The data were tested for normality using the Shapiro‐Wilk test. Differences between more than two groups were analysed using one‐way anova, and the differences between two groups were analysed using the *t*‐test, all of which were in parametric distribution. The Bonferroni test was used as post‐hoc analysis after the one‐way anova test. Differences between more than two groups were analysed using the Kruskal‐Wallis test and differences between two groups were analysed using the Mann‐Whitney test—all in non‐parametric distribution. Dunn's multiple comparisons test was used as post‐hoc analysis after Kruskal‐Wallis test. A neoplastic index (NI) was developed by binary logistic regression based on the ECPKA‐Ab and CRP levels. The statistical program, SPSS for Windows version 23 (IBM Corp., Armonk, New York), was used to create a multivariable equation using ECPKA‐Ab and CRP as the individual variables and the NI as the result. The cut‐off value for discrimination between the two groups was estimated by analysing the receiver‐operating characteristic (ROC) curve. All graphs are presented as box and whisker plots. All the data are shown as the median and range obtained in at least three independent experiments. The statistical analyses were performed using SPSS and GraphPad Prism version 7 (GraphPad Software, Inc., La Jolla, California). A *P*‐value of <0.05 was considered statistically significant.

## 3 RESULTS

We tested serum samples from 167 dogs with a diagnosis of cancer and 320 dogs without a diagnosis of cancer, including those that were considered to be healthy or with a benign tumour or non‐tumour disease. The signalment data are summarized in Table 1. The median age of the total study population was 10.00 (range: 0.25‐18.00) years and the main breeds were Maltese (n = 95) and Shih‐tzu (n = 71). The cancers were categorized according to the cell of origin—carcinoma, sarcoma, haematopoietic lymphoreticular or neuroendocrine. The final diagnoses in the group of dogs with cancer (n = 167) are shown in Table 2. The cancer group included 98 dogs with carcinoma (malignant mammary gland tumour [MMGT], transitional cell carcinoma [TCC], hepatocellular carcinoma [HCC], pulmonary adenocarcinoma [PAC]), 33 with sarcoma (melanoma [Mel], hemangiosarcoma [HSA] and soft tissue sarcoma), 35 with haematopoietic/lymphoreticular disease (lymphoma and leukaemia), and one with a neuroendocrine tumour (pheochromocytoma). The three dogs with leukaemia had either chronic lymphocytic leukaemia (n = 2) or acute megakaryocytic leukaemia (n = 1). The diseases in the 155 dogs in the non‐tumour group are categorized according to organ system in Table 3. The most common diseases were cardiovascular (n = 42) and urologic (n = 30).

**Table 1:** Signalment data for dogs included in the present study

	Normal	Non‐tumour diseases	Benign tumour	Malignant tumour
n	123	155	42	167
Median age (range)	3.00 (0.25‐16.00)	10.92 (0.25‐18.00)	10.46 (0.50‐16.70)	13.00 (0.59‐18.00)
Sex (n)	CM (25), F (47), M (29), SF (22)	CM (67), F (18), M (9), SF (61)	CM (18), F (11), M (1), SF (12)	CM (64), F(24), M (3), SF (76)
Breed (n)	Beagle (60), Miniature poodle (15), Maltese (10), Pomeranian (7), Bichon Frise (5), other (26)	Maltese (46), Shih‐tzu (19), Miniature poodle (17), Mongrel (12), Miniature schnauzer (11), Cocker spaniel (10), Yorkshire terrier (10), other (30)	Cocker spaniel (10), Maltese (8), Shih‐tzu (4), Miniature Poodle (4), other (16)	Shih‐tzu (47), Maltese (29), Cocker Spaniel (25), Mongrel (12) Yorkshire terrier (11), other (43)

Abbreviations: CM, castrated male; F, female; M, male; SF, spayed female.

**Table 2:** Types of cancer and numbers of affected dogs

Type of cancer (cell origin)	Cancer (n)	Total (n)
Carcinoma (epithelial)	Malignant MGT (29), TCC (19), HCC (9), pulmonary adenocarcinoma (6), RCC (4), adenocarcinoma of the prostate (4), ASAC (3), SCC (3), other (21)	98
Sarcoma (mesenchymal)	Hemangiosarcoma (9), melanoma (9), soft tissue sarcoma (4), other (10)	33
Haematopoietic or lymphoreticular	Lymphoma (28), leukaemia (3), other (4)	35
Neuroendocrine	Pheochromocytoma (1)	1

Abbreviations: ASAC, anal sac adenocarcinoma; HCC, hepatic cellular carcinoma; MGT, mammary gland tumour; RCC, renal cell carcinoma; SCC, squamous cell carcinoma;TCC, transitional cell carcinoma.

**Table 3:** Disease types and numbers of dogs with non‐tumour diseases in the present study

Types of non‐tumour disease	n
Cardiovascular	42
Urologic	30
Gastrointestinal	25
Immune‐mediated	25
Neurologic	21
Endocrine	20
Hepatobiliary	13
Dermatologic	12
Infectious	9
Musculoskeletal	7
Respiratory	5

**Figure 1:**
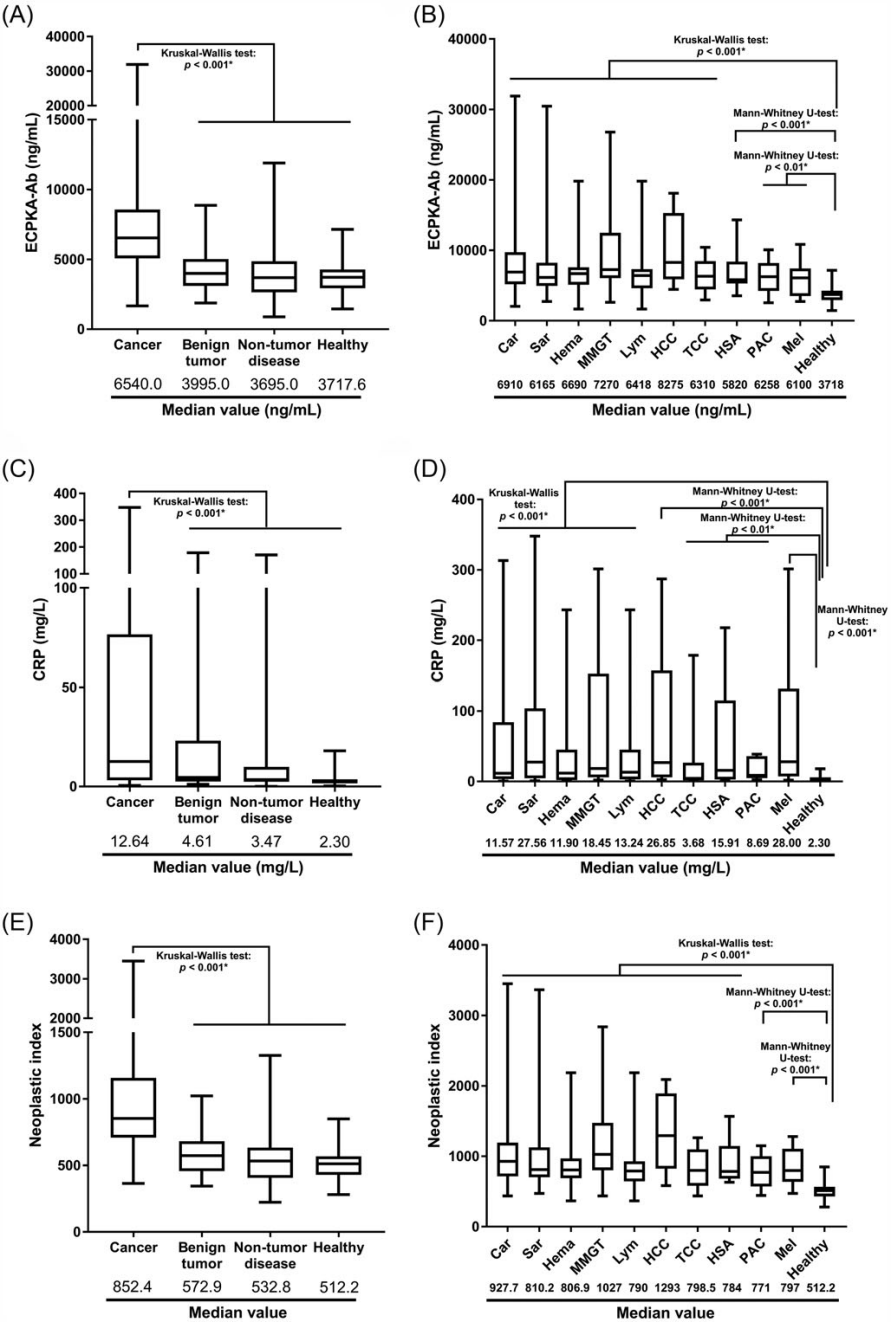
Enzyme‐linked immunosorbent assay data for the ECPKA autoantibody, CRP and NI values in each study group. A, ECPKA‐Ab levels in the cancer, benign tumour, non‐tumour disease and healthy study groups. B, ECPKA‐Ab levels in the different cancer subgroups, including carcinoma, sarcoma, haematopoietic/lymphoreticular disease and the subcategories therein. C, CRP levels in the cancer, benign tumour, non‐tumour disease, and healthy study groups. D, CRP levels in the different cancer subgroups, including carcinoma, sarcoma, haematopoietic/lymphoreticular disease and the subcategories therein. E, The NI value in the cancer, benign tumour, non‐tumour disease and healthy study groups. (F) the NI value in the different cancer subgroups, including carcinoma, sarcoma, haematopoietic/lymphoreticular disease and the subcategories therein. All graphs are shown as box and whisker plots. Each box includes the interquartile range; the line within each box represents the median and the whiskers represent the range, extending to a maximum of 1.5 times the interquartile range. Ab, antibodies; Car, carcinoma; CRP, C‐reactive protein; ECPKA, extracellular cyclic AMP‐dependent protein kinase; HCC, hepatocellular carcinoma; Hema, haematopoietic/lymphoreticular; HSA, hemangiosarcoma; Lym, lymphoma; Mel, malignant melanoma; MMGT, malignant mammary gland tumour; NI, neoplastic index; Sar, sarcoma; TCC, transitional cell carcinoma

The serum ECPKA‐Ab level was analysed by ELISA (Figure 1). The median ECPKA‐Ab levels in the cancer, benign tumour, non‐tumour disease, and healthy control groups were 6540.0 (range: 1665‐31 900) ng/mM, 3995.0 (range: 1875‐8870) ng/mL, 3695.0 (range: 890‐11 900) ng/mL, and 3717.6 (range: 1455‐7153) ng/mL, respectively. The median ECPKA‐Ab level in the cancer group was significantly higher than the levels in the benign tumour, non‐tumour disease and healthy groups (all *P* < 0.0001, Kruskal‐Wallis test). The median ECPKA‐Ab levels in the dogs with carcinoma, sarcoma and haematopoietic/lymphoreticular disease were 6910 (range 2040‐31 900) ng/mL, 6165 (range 2720‐30 460) ng/mL, and 6690 (range 1665‐19 820), respectively, and the level in the dog with the neuroendocrine tumour was 6440 ng/mL. Higher median ECPKA‐Ab levels were detected in the dogs with diagnoses of MMGT (7270 ng/mL), lymphoma (6418 ng/mL), HCC (8275) ng/mL, TCC (6310 ng/mL), HSA (5820 ng/mL), PAC (6258 ng/mL), and Mel (6100 ng/mL).Dog breed did not appear to have a significant effect on the ECPKA‐Ab level in dogs with or without cancer (*P* = 0.621 and *P* = 0.204, respectively). There was no significant difference between male and female dogs in both the cancer and non‐cancer groups (*P* = 0.557 and *P* = 0.624, respectively). To analyse the effect of neutralization, ECPKA‐Ab levels were compared in both castrated and non‐castrated male dogs and in both spayed and non‐spayed female dogs, respectively. In the dogs with cancer, there was no significant difference between spayed and non‐spayed female dogs in terms of ECPKA‐Ab levels (*P* = 0.08); the number of male dogs in the cancer group was too small to analyse the effect of castration. In the non‐cancer dogs, there was no difference between spayed and non‐spayed female dogs (*P* = 0.128) or between the castrated and non‐castrated male dogs (*P* = 0.778) in terms of ECPKA‐Ab levels.

The CRP levels in the canine serum samples were analysed by ELISA (Figure 1). The median CRP levels in the cancer, benign tumour, non‐tumour disease, and healthy groups were 12.64 (range: 0.5‐348) mg/L, 4.61 (range: 1.1‐178.8) mg/L, 3.47 (range: 0‐170.4) mg/L and 2.3 (range: 0.27‐18.03) mg/L, respectively. The CRP level in the cancer group was significantly higher than the levels in the benign tumour, non‐tumour disease and healthy groups (all *P* < 0.0001, Kruskal‐Wallis test). The median CRP levels in the carcinoma, sarcoma and haematopoietic/lymphoreticular disease subgroups were 11.57 (range: 0.52‐313.3) mg/L, 27.56 (range: 1‐348) mg/L, and 11.9 (range: 0.5‐243) mg/L, respectively; the CRP level in the dog with the neuroendocrine tumour was 0.9 mg/L. Higher CRP levels were detected in the dogs with diagnoses of MMGT (18.45 mg/L), lymphoma (13.24 mg/L), HCC (26.85 mg/L), TCC (3.68 mg/L), HSA (15.91 mg/L), PAC (8.69 mg/L), and Mel (28 mg/L).

The NI derived from the ECPKA‐Ab and CRP levels is shown in Figure 1E, F. The NI was higher in the cancer group than in the benign tumour, non‐tumour disease and healthy study groups (all *P* < 0.001, Kruskal‐Wallis test). The subcategories in the cancer group had significantly high NI values (Figure 1F).

**Figure 2:**
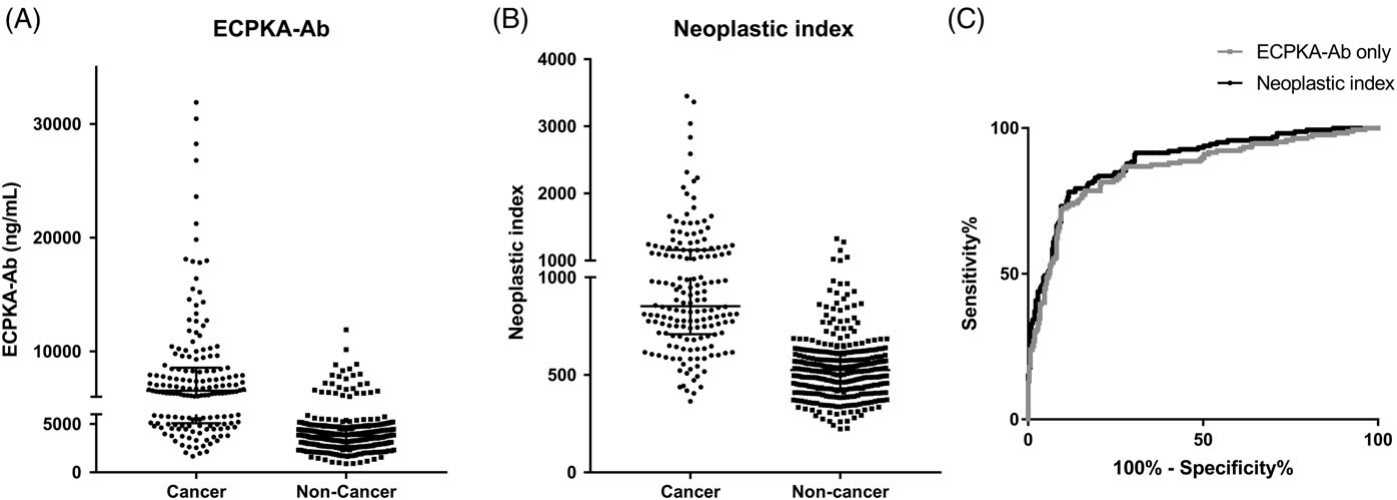
Differences in the ECPKA‐Ab level and NI value between dogs with and without a diagnosis of cancer. A, ECPKA‐Ab levels. B, NI levels. C, Receiver‐operating characteristic curve for ECPKA‐Ab and NI. All error bars represented in this figure are shown as median with interquartile range. Ab, antibodies; ECPKA, extracellular cyclic AMP‐dependent protein kinase; NI, neoplastic index

A ROC analysis was then performed to evaluate the value of ECPKA‐Ab and NI as diagnostic biomarkers of cancer when all dogs in the study population were classified according to whether they did or did not have cancer (Figure 2). The non‐cancer group included the benign tumour, non‐tumour disease and healthy study groups. Both the ECPKA‐Ab level and the NI value were significantly higher in the cancer group (both *P* < 0.001, Mann‐Whitney *U*‐test). The area under the receiver‐operating characteristic curve (AUROC) was 0.86 for the ECPKA‐Ab level and 0.89 for the NI value. The diagnostic accuracy of the combination of ECPKA‐Ab and NI is shown in Table 4.

**Table 4:** Diagnostic ability of ECPKA‐ab and neoplastic index

	ECPKA‐ab	NI
AUROC	0.86	0.89
95% cl	0.82‐0.90	0.85‐0.92
*P*‐value	<0.0001	<0.0001
Sensitivity	80.84	82.93
Specificity	79.38	80.88
Accuracy	79.88	81.57
PPV	67.16	69.04
NPV	88.81	90.21

Abbreviations: AUROC, area under the receiver‐operating characteristic curve; ECPKA‐Ab, extracellular cyclic AMP‐dependent protein kinase autoantibodies; NI, neoplastic index; NPV, negative predictive value; PPV, positive predictive value.

**Figure 3:**
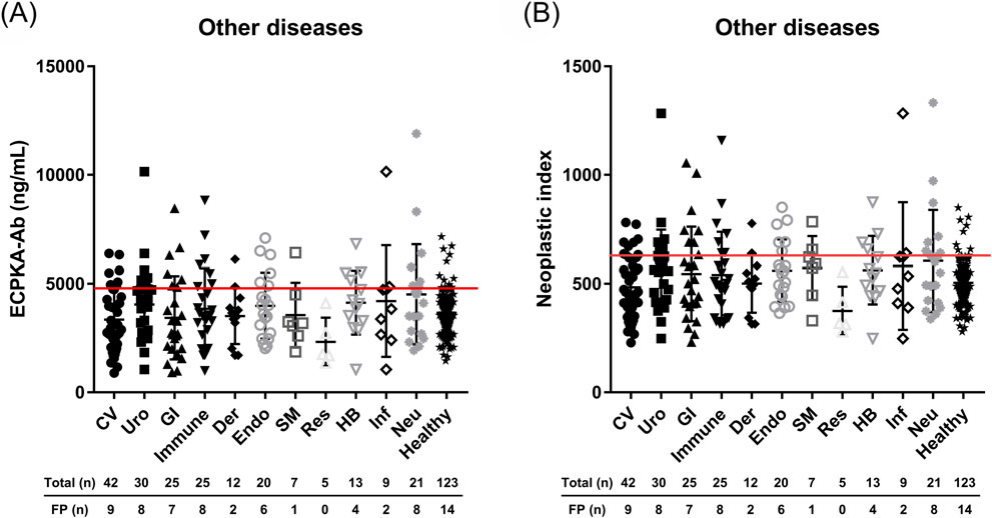
Distribution of ECPKA‐Ab and NI when the diseases were classified according to organ system. A scatter plot of other disease group and healthy group was shown in case of A, ECPKA‐Ab levels and B, NI value. The data are shown as the mean and SD. The number of dogs are shown as footnotes. Ab, antibodies; CV, cardiovascular; Der, dermatologic; ECPKA, extracellular cyclic AMP‐dependent protein kinase; Endo, endocrine; FP, false positive; GI, gastrointestinal; HB, hepatobiliary; immune, immune‐mediated; Inf, infectious; Neu, neurologic; NI, neoplastic index; Res, respiratory; SM, skeletomuscular; Uro, urologic [Colour figure can be viewed at wileyonlinelibrary.com]

To determine whether the ECPKA‐Ab level or NI increases in certain diseases, the values for these parameters were analysed by type of disease (Figure 3); there was no significant difference in either of these values between any of the disease groups and the healthy group (Kruskal‐Wallis test with Dunn's multiple comparisons test, *P* = 0.1395).

## 4 DISCUSSION

The purpose of this study was to determine the presence and level of ECPKA‐Ab in canine serum and to evaluate the value of ECPKA‐Ab as a biomarker of canine cancer. ECPKA‐Ab were detected in canine serum and at higher levels in the cancer group than in the non‐cancer group. The CRP level was also increased in the cancer group, and the NI, developed by regression analysis of both ECPKA‐Ab and CRP, had a higher AUROC than ECPKA‐Ab alone when both NI and ECPKA‐Ab had an AUROC >0.85.

The finding of a significantly higher serum ECPKA‐Ab level in dogs with cancer in our present study (Figures 1 and 2) is consistent with our previous findings in dogs.^9^ Moreover, when the cancers were classified, as shown in Table 2, the ECPKA‐Ab level was found to be increased regardless of the cell of origin (Figure 1B) and was significantly higher in the cancer group than in the healthy group; this finding has also been reported in human patients.^24^ There are many other studies in the human literature that report the ECPKA‐Ab level is increased in patients with various types of cancer.^25^, ^26^, ^27^ Therefore, the ECPKA autoantibody is regarded as a universal cancer biomarker.^27^ Whereas other specific biomarkers can detect only one specific type of cancer, for example, prostate‐specific antigen for prostate cancer and α‐fetoprotein for liver cancer,^27^ ECPKA‐Ab can be used to detect various types of malignancies. Furthermore, based on our findings, it may be possible for ECPKA‐Ab to be used as a screening marker for occult cancer, considering that cancer in most dogs progresses subclinically and frequently goes undetected using conventional blood tests. It is ideal for the screening biomarker used to diagnose occulting cancer to have a high sensitivity >90%, but considering that most dogs with cancer are diagnosed at the late stages—when clinical symptoms have begun to manifest—this marker could be a clinically meaningful biomarker. Additionally, the ECPKA‐Ab level can be used to help predict the risk of malignancy in dogs with masses already detected by physical examination or imaging, as this level is not increased in the serum of dogs with benign tumours (Figure 1). Biopsy is the standard procedure for confirming whether a mass is cancerous or benign, however, it has several limitations in clinical use, including issues with anaesthesia, haemorrhage in coagulopathy, and accessibility. Cytology is a simple diagnostic method that is used clinically, and although it has a high specificity, the sensitivity is low. Therefore, ECPKA‐Ab levels can provide additional information to determine whether the mass is malignant or benign.

In the present study, we did not compare the ECPKA and ECPKA‐Ab levels within the same samples, so it was not possible to compare the diagnostic accuracy of ECPKA with that of ECPKA‐Ab. However, given the findings of studies in humans,^10^, ^24^ the diagnostic accuracy of ECPKA‐Ab would be expected to be better than that of ECPKA in dogs. The diagnostic accuracy achieved using autoantibodies of several biomarkers of cancer, for example, α‐fetoprotein (liver cancer) and CA125 (ovarian cancer), has been compared with that of the biomarkers alone in the detection of cancer.^24^ The presence of ECPKA‐Ab in human serum has been confirmed, and its usefulness in the diagnosis of human cancer has been suggested.^24^, ^25^, ^26^

In the present study, the NI had a higher AUROC and higher sensitivity, specificity, and accuracy than ECPKA‐Ab alone (Figure 2, Table 4), indicating that ECPKA‐Ab is a more powerful biomarker of cancer when combined with CRP. This result is very similar to the findings for thymidine kinase 1 activity reported by Selting et al.^21^, ^22^ In their studies, thymidine kinase 1 activity was used as a serum biomarker of cancer in dogs, and its diagnostic accuracy was improved when used with CRP. As in other studies that have demonstrated an increased CRP level in dogs with cancer,^17^, ^18^ we found a significantly higher CRP level in dogs with cancer than in those without cancer (Figure 1(C) (D)). However, because CRP shows a nonspecific increase in various situations, including inflammation,^18^ it could not be used as a sole biomarker for cancer. Although elevated CRP is not in itself a biomarker of cancer, it may serve as a co‐factor to increase the diagnostic accuracy of other biomarkers in detecting cancer.

The AUROC of both ECPKA‐Ab and NI were 0.86 and 0.89, respectively. At the best cut‐off point, which is determined as the point where the sum of sensitivity and specificity is highest (at the same time, when sensitivity is greater than specificity), positive predictive value (PPV) was rather low in both ECPKA‐Ab and NI (Table 4). Although positive and negative predictive values (NPV) are changeable by included data, these markers have a relatively low PPV and a high NPV. This result is based on setting the cut‐off point to where the sensitivity is high. These predictive values suggest that ECPKA‐Ab and NI could be used as a rule‐in biomarker, but not a rule‐out biomarker. Similarly, bladder tumour antigen (BTA), a urine biomarker for transitional cell carcinoma (TCC), has a higher sensitivity (85‐90%) and a much lower specificity (35‐94.4%)^28^, ^29^; therefore, it can rule‐in TCC, but not rule‐out TCC. The low specificity of BTA results in the inability to distinguish bladder cancer from bacterial cystitis or haemorrhagic cystitis.^28^, ^29^ On the other hand, the TK1 activity test and B‐raf proto‐oncogene serine/threonine kinase gene mutation test have high specificities and low sensitivities, suggesting that it can be used to confirm malignant tumours.^21^, ^22^, ^23^, ^30^ A high sensitivity test is suitable as a screening tool, and depending on the result, additional tests may be needed to confirm the diagnosis. High‐specificity tests can confirm cancer, but they cannot diagnose cancer if the result is negative.

To determine whether a specific variable other than cancer could lead to a high ECPKA‐Ab level in canine serum, we analysed the effect of sex, castration stratus, breed and type of non‐tumour disease. We found no significant effect of sex, castration status, or breed in this study. However, only a few breeds and a small number of dogs per breed were included in this investigation; thus, it is possible that another result could be obtained in a different study sample with different breeds. Furthermore, there was no significant variation in the ECPKA‐Ab level according to type of disease in the non‐tumour disease group (Figure 3). However, the spectrum of disease included in this patient group was not necessarily representative, so the ECPKA‐Ab level should be further investigated in various diseases. In particular, immune‐mediated diseases that result from self‐perpetuating B‐cells and autoantibodies^31^ need to be studied in more detail to assess their potential effect on the ECPKA‐Ab level. Moreover, the main immune‐mediated disease in the present study was atopic dermatitis with lesser numbers of patients with immune‐mediated haemolytic anaemia or thrombocytopenia. Studies that include larger numbers of patients with these and other autoimmune diseases are needed.

The causes of false‐negative canine cases, that is, dogs with cancer and low ECPKA‐Ab levels, also need to be investigated. ECPKA‐Ab consists mainly of immunoglobulin G, which has antibody‐forming ability in the body and could affect the ECPKA‐Ab level. It is presumed that if a dog with cancer is immunosuppressed, its ability to form antibodies would decrease and the ECPKA‐Ab level would be low. Furthermore, follow‐up investigations are required to confirm the relationship between occult cancer and survival. Additional studies are also needed to determine whether the ECPKA‐Ab level and the NI value can be used for therapeutic monitoring.

In summary, we have shown that the ECPKA‐Ab level and the NI value are meaningful serum biomarkers of canine cancer. In the present study, dogs with cancer had higher ECPKA‐Ab levels than those without cancer and a combination of the ECPKA‐Ab and CRP levels had increased the diagnostic accuracy for detecting cancer. Therefore, this combination of biomarkers may help to improve the efficacy of cancer treatment by increasing the diagnosis rate—even in dogs with cancer that appear clinically healthy.

## CONFLICT OF INTEREST

None of the other authors of this paper has a financial or personal relationship with other persons or organizations that could inappropriately influence or bias the content of the paper.

## References

[1] Allemani C , Weir HK , Carreira H , et al. Global surveillance of cancer survival 1995–2009: analysis of individual data for 25 676 887 patients from 279 population‐based registries in 67 countries (CONCORD‐2). Lancet. 2015;385:977‐1010. 25467588 10.1016/S0140-6736(14)62038-9 PMC4588097

[2] Siegel RL , Miller KD , Jemal A . Cancer statistics, 2016. CA Cancer J Clin. 2016;66:7‐30. 26742998 10.3322/caac.21332

[3] MacEwen EG . Spontaneous tumors in dogs and cats: models for the study of cancer biology and treatment. Cancer Metastasis Rev. 1990;9:125‐136. 2253312 10.1007/BF00046339

[4] Kotani T . Chapter 12Protein kinase a activity and hedgehog signaling pathway In: Litwack G, ed. Vitamins & Hormones. Vol 88 Cambridge, Massachusetts: Academic Press; 2012:273‐291. 22391308 10.1016/B978-0-12-394622-5.00012-2

[5] Cho YS , Park YG , Lee YN , et al. Extracellular protein kinase A as a cancer biomarker: its expression by tumor cells and reversal by a myristate‐lacking Cα and RIIβ subunit overexpression. Proc Natl Acad Sci U S A. 2000;97:835‐840. 10639166 10.1073/pnas.97.2.835 PMC15417

[6] Cho YS , Lee YN , Cho‐Chung YS . Biochemical characterization of extracellular cAMP‐dependent protein kinase as a tumor marker. Biochem Biophys Res Commun. 2000;278:679‐684. 11095968 10.1006/bbrc.2000.3853

[7] Kita T , Goydos J , Reitman E , et al. Extracellular cAMP‐dependent protein kinase (ECPKA) in melanoma. Cancer Lett. 2004;208:187‐191. 15142677 10.1016/j.canlet.2004.02.018

[8] Wang H , Li M , Lin W , et al. Extracellular activity of cyclic AMP–dependent protein kinase as a biomarker for human cancer detection: distribution characteristics in a normal population and cancer patients. Cancer Epidemiol Biomarkers Prev. 2007;16:789‐795. 17416772 10.1158/1055-9965.EPI-06-0367

[9] Bhang DH , Choi US , Kim BG , et al. Characteristics of extracellular cyclic AMP‐dependent protein kinase as a biomarker of cancer in dogs. Vet Comp Oncol. 2017;15:1585‐1589. 28185388 10.1111/vco.12304

[10] Nesterova MV , Johnson N , Cheadle C , et al. Autoantibody cancer biomarker: extracellular protein kinase a. Cancer Res. 2006;66:8971‐8974. 16982736 10.1158/0008-5472.CAN-06-1049

[11] Weinstein P , Skinner M , Sipe J , Lokich J , Zamcheck N , Cohen A . Acute‐phase proteins or tumour markers: the role of SAA, SAP, CRP and CEA as indicators of metastasis in a broad spectrum of neoplastic diseases. Scand J Immunol. 1984;19:193‐198. 6200925 10.1111/j.1365-3083.1984.tb00919.x

[12] Toniatti C , Arcone R , Majello B , Ganter U , Arpaia G , Ciliberto G . Regulation of the human C‐reactive protein gene, a major marker of inflammation and cancer. Mol Bio Med. 1990;7:199‐212. 2170808

[13] Erlinger TP , Platz EA , Rifai N , Helzlsouer KJ . C‐reactive protein and the risk of incident colorectal cancer. JAMA. 2004;291:585‐590. 14762037 10.1001/jama.291.5.585

[14] Pine SR , Mechanic LE , Enewold L , et al. Increased levels of circulating interleukin 6, interleukin 8, C‐reactive protein, and risk of lung cancer. J Natl Cancer Inst. 2011;103:1112‐1122. 21685357 10.1093/jnci/djr216 PMC3139587

[15] Mahmoud FA , Rivera NI . The role of C‐reactive protein as a prognostic indicator in advanced cancer. Curr Oncol Rep. 2002;4:250‐255. 11937016 10.1007/s11912-002-0023-1

[16] Siemes C , Visser LE , J‐WW C , et al. C‐reactive protein levels, variation in the C‐reactive protein gene, and cancer risk: the Rotterdam study. J Clin Oncol. 2006;24:5216‐5222. 17114654 10.1200/JCO.2006.07.1381

[17] Mischke R , Waterston M , Eckersall PD . Changes in C‐reactive protein and haptoglobin in dogs with lymphatic neoplasia. Vet J. 2007;174:188‐192. 16901733 10.1016/j.tvjl.2006.05.018

[18] Nakamura M , Takahashi M , Ohno K , et al. C‐reactive protein concentration in dogs with various diseases. J Vet Med Sci. 2008;70:127‐131. 18319571 10.1292/jvms.70.127

[19] Planellas M , Bassols A , Siracusa C , et al. Evaluation of serum haptoglobin and C‐reactive protein in dogs with mammary tumors. Vet Clin Pathol. 2009;38:348‐352. 19392756 10.1111/j.1939-165X.2009.00139.x

[20] Nielsen L , Toft N , Eckersall PD , Mellor DJ , Morris JS . Serum C‐reactive protein concentration as an indicator of remission status in dogs with multicentric lymphoma. J Vet Intern Med. 2007;21:1231‐1236. 18196731 10.1892/07-058.1

[21] Selting KA , Sharp CR , Ringold R , Knouse J . Serum thymidine kinase 1 and C‐reactive protein as biomarkers for screening clinically healthy dogs for occult disease. Vet Comp Oncol. 2015;13:373‐384. 23859156 10.1111/vco.12052

[22] Selting KA , Ringold R , Husbands B , Pithua PO . Thymidine kinase type 1 and C‐reactive protein concentrations in dogs with spontaneously occurring cancer. J Vet Intern Med. 2016;30:1159‐1166. 27214230 10.1111/jvim.13954 PMC5089580

[23] Thamm DH , Kamstock DA , Sharp CR , et al. Elevated serum thymidine kinase activity in canine splenic hemangiosarcoma. Vet Comp Oncol. 2012;10:292‐302. 22236280 10.1111/j.1476-5829.2011.00298.x

[24] Nesterova M , Johnson N , Cheadle C , Cho‐Chung YS . Autoantibody biomarker opens a new gateway for cancer diagnosis. Biochim Biophys Acta. 2006;1762:398‐403. 16483750 10.1016/j.bbadis.2005.12.010

[25] Choi CW , Seo HY , Sung HJ , et al. Detection of extracellular protein kinase a (ECPKA) autoantibody in non‐hodgkin's lymphoma: its increased antibody titer decreases with a favorable treatment response. Blood. 2006;108:4621‐4621.

[26] Loilome W , Yooyuen S , Namwat N , et al. PRKAR1A overexpression is associated with increased ECPKA autoantibody in liver fluke‐associated cholangiocarcinoma: application for assessment of the risk group. Tumor Biol. 2012;33:2289‐2298. 10.1007/s13277-012-0491-3 22922884

[27] Zaenker P , Ziman MR . Serologic autoantibodies as diagnostic cancer biomarkers—a review. Cancer Epidemiol Biomarkers Prev. 2013;22:2161‐2181. 24057574 10.1158/1055-9965.EPI-13-0621

[28] Henry CJ , Tyler JW , McEntee MC , et al. Evaluation of a bladder tumor antigen test as a screening test for transitional cell carcinoma of the lower urinary tract in dogs. Am J Vet Res. 2003;64:1017‐1020. 12926595 10.2460/ajvr.2003.64.1017

[29] Billet J‐PHG , Moore AH , Holt PE . Evaluation of a bladder tumor antigen test for the diagnosis of lower urinary tract malignancies in dogs. Am J Vet Res. 2002;63:370‐373. 11926180 10.2460/ajvr.2002.63.370

[30] Mochizuki H , Shapiro SG , Breen M . Detection of BRAF mutation in urine DNA as a molecular diagnostic for canine urothelial and prostatic carcinoma. PLoS One. 2015;10:e0144170. 10.1371/journal.pone.0144170 PMC4674145 26649430

[31] Edwards J , Cambridge G , Abrahams V . Do self‐perpetuating B lymphocytes drive human autoimmune disease? Immunology. 1999;97:188‐196. 10447731 10.1046/j.1365-2567.1999.00772.x PMC2326840

